# Veno-Arterial Extracorporeal Membrane Oxygenation for Treating Refractory Shock in Severe Metformin-Associated Lactic Acidosis: A Case Report

**DOI:** 10.7759/cureus.81197

**Published:** 2025-03-25

**Authors:** Kisei Sai, Naoya Miura, Asuka Tsuchiya, Seiji Morita, Yoshihide Nakagawa

**Affiliations:** 1 Department of Emergency and Critical Care Medicine, Tokai University School of Medicine, Isehara, JPN

**Keywords:** continuous renal replacement therapy (crrt), metformin-associated lactic acidosis, refractory shock, severe acidemia, veno-arterial extracorporeal membrane oxygenation (va ecmo)

## Abstract

Metformin-associated lactic acidosis (MALA) is a rare but life-threatening complication with mortality rates exceeding 10-30%. While renal replacement therapy (RRT) remains the cornerstone of treatment, mechanical circulatory support may be necessary in cases complicated by severe cardiovascular dysfunction. We report a case of severe MALA with unprecedented metabolic derangement successfully treated with veno-arterial extracorporeal membrane oxygenation (VA-ECMO) and continuous renal replacement therapy (CRRT). A 39-year-old male with type 2 diabetes mellitus presented with altered mental status following influenza A infection. Initial assessment revealed severe metabolic acidosis (pH 6.355) with markedly elevated lactate (52.7 mmol/L), acute kidney injury, and cardiovascular collapse. Echocardiography demonstrated severe left ventricular dysfunction with an ejection fraction below 20%. Despite initial resuscitation efforts, including high-dose vasopressors, the patient developed cardiac arrest with pulseless electrical activity, requiring cardiopulmonary resuscitation. After the return of spontaneous circulation, VA-ECMO was initiated for refractory shock unresponsive to high-dose vasopressors. MALA was diagnosed based on the clinical presentation and medication history, although metformin levels could not be measured due to assay unavailability. Under combined VA-ECMO and CRRT support, the patient's metabolic parameters improved steadily, with a gradual decrease in lactate levels and an improvement in pH. Left ventricular function recovered significantly, allowing VA-ECMO discontinuation after 26 hours. The patient was successfully weaned from mechanical ventilation with subsequent rehabilitation, achieving discharge with intact cognitive function and no neurological sequelae. By this time, both cardiac and renal functions had normalized. This case demonstrates the successful use of mechanical circulatory support in extreme metabolic derangement and illustrates how viral illnesses can precipitate severe MALA through acute kidney injury. The successful outcome suggests that early recognition and aggressive intervention with combined VA-ECMO and CRRT might be beneficial in selected patients with MALA-induced cardiovascular collapse. This case also highlights the importance of considering MALA in unexplained severe lactic acidosis, particularly in diabetic patients during acute illness.

## Introduction

Metformin is widely prescribed as an oral hypoglycemic agent that improves insulin sensitivity and suppresses glucose production in the liver. Its safety and cardiovascular benefits are well-established [1]. However, metformin-associated lactic acidosis (MALA) is a rare but life-threatening condition with an annual incidence of 3 to 10 cases per 100,000 person-years among metformin-treated diabetic patients identified through hospital-based studies. [2,3]. Despite its low incidence and the mortality of MALA differing among studies, MALA has a significant mortality rate of more than 10-30 % [2,4,5]. To reduce mortality, renal replacement therapy (RRT) has been the cornerstone for treating severe MALA, primarily through drug removal and correction of metabolic acidosis [6].

The pathophysiology of severe MALA involves complex cardiovascular effects, including direct myocardial depression from metformin accumulation [7] and profound acidosis-induced cardiac dysfunction [8]. Metformin accumulation, even at therapeutic doses when clearance is impaired, can disrupt mitochondrial function by inhibiting complex I of the electron transport chain, resulting in decreased ATP production and compromised myocardial contractility. Simultaneously, severe acidosis compromises cardiac function by reducing myofilament calcium sensitivity and altering calcium-force relationships in cardiac muscle cells. In such cases, conventional treatment with RRT alone may be insufficient to overcome refractory shock. Although veno-arterial extracorporeal membrane oxygenation (VA-ECMO) can provide essential hemodynamic support during this critical period, clinical studies on mechanical circulatory support in severe MALA remain limited. VA-ECMO may potentially offer benefits beyond hemodynamic support, such as immediate restoration of systemic perfusion and maintenance of circulatory stability for effective CRRT function.

Here, we report a case of severe MALA with refractory shock and cardiac arrest successfully treated with RRT combined with VA-ECMO, which provides insights into the potential role of mechanical circulatory support in managing this critical condition.

## Case presentation

A 39-year-old male (height 180 cm, weight 87.2 kg, BMI 26.9) presented in an unconscious state to our emergency department. He had recently been diagnosed with influenza A and, hence, had stopped working. On presentation, the patient was comatose (Glasgow Coma Scale score, E1V1M1). His blood pressure was unmeasurable owing to barely palpable carotid pulses. He exhibited an axillary temperature of 31.2°C and severe hypoxemia with SpO_2_ of 74% despite receiving 10 L of oxygen. After intubation and artificial ventilation, initial arterial blood gas analysis revealed severe metabolic acidosis: pH 6.355, base excess −42.8 mmol/L, and a markedly elevated lactate level (52.7 mmol/L). Laboratory tests revealed significant abnormalities, including leukocytosis, acute kidney injury (blood urea nitrogen 26 mg/d and creatinine 5.15 mg/dL), elevated liver enzyme, and hyperglycemia (Table 1).

**Table 1:** Laboratory data on admission pH: power of hydrogen; PaCO 2: partial pressure of arterial carbon dioxide; PaO2: partial pressure of arterial oxygen; HCO3: hydrogen carbonate; BE: base excess;WBC: white blood cell; RBC: red blood cell; Hb: hemoglobin; Plt: platelet; Alb: albumin; CK: creatine kinase; AST: aspartate aminotransferase; ALT: alanine aminotransferase; T.bil: total bilirubin; BUN: blood urea nitrogen; Cre: creatinine;Na: sodium; K: potassium; Cl: chloride; CRP: C-reactive protein; HbA1c: glycated hemoglobin *Arterial blood gas values obtained on FiO_2_ 1.0.

Test	Result	Units	Normal range of value
Blood gas analysis*			
PH	6.355		7.380–7.460
PaCO_2_	38.8	Torr	35.0–45.0
PaO_2_	540	Torr	75.0–100.0
HCO_3-_	2.2	mmol/L	21.0–28.0
Base excess	-42.8	mmol/L	-2.0–2.0
Lactic acid	52.7	mmol/L	0.56–1.39
Blood cell count			
WBC	54,400	/μL	3,300–8,600
RBC	5.17×10^6^	/μL	4.35–4.92×10^6^
Hb	15.6	g/dl	11.6–14.8
Plt	54.9×10^4^	/μL	15.8–34.8×10^4^
Biochemistry			
Alb	4.4	g/dl	4.1–5.1
CK	495	U/L	59–248
AST	88	U/L	13–30
ALT	73	U/L	10–42
LDH	770	U/L	124–222
T-bil	0.2	mg/dl	2.7–4.6
Cr	5.15	mg/dl	0.65–1.07
BUN	26	mg/dl	8–20
Na	148	mEq/L	138–145
K	5.8	mEq/L	3.6–4.8
Cl	102	mEq/L	101–108
CRP	4.02	mg/dl	0.00–0.14
HbA1c	6.5	%	4.9–6.0

**Figure 1:**
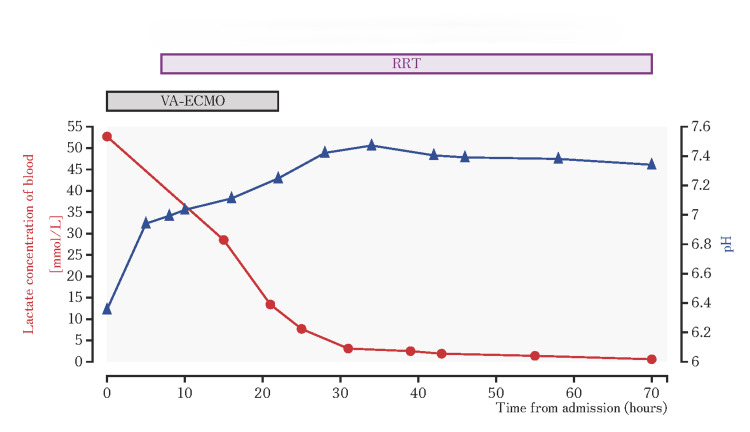
Temporal variation in the lactate concentration and pH after admission

Notably, severe left ventricular dysfunction with diffuse hypokinesia and an ejection fraction below 20% was detected via echocardiography. Further echocardiographic assessment revealed decreased right ventricular function, reflecting the global hypokinesis, while no specific diastolic dysfunction was observed. Initial resuscitation included fluid resuscitation with extracellular fluid, bicarbonate therapy with 250 ml of 7% bicarbonate, and dopamine administration (20 μg/kg/min). However, 35 minutes after arrival, the patient developed cardiac arrest with pulseless electrical activity. After cardiopulmonary resuscitation continued for two minutes, spontaneous circulation was restored. Noradrenaline was added, but persistent circulatory failure necessitated the initiation of VA-ECMO. After stabilization using VA-ECMO, further medical history of the patient was revealed; he had type 2 diabetes mellitus treated with metformin 500 mg daily. Based on this information and the severe lactic acidosis, MALA was diagnosed, and CRRT was promptly initiated for metformin removal and metabolic correction. Although we considered alternative causes of severe lactic acidosis, including sepsis and shock-induced tissue hypoxia, the extreme elevation of lactate could not be adequately explained by these mechanisms alone. Toxicology screening did not detect other substances that could account for this degree of metabolic derangement. This combined support gradually improved the patient's metabolic derangement (Figure 1).

Lactate levels decreased from the initial concentration of 52.7 mmol/L with a concurrent improvement in pH, and left ventricular function improved significantly, with ejection fraction recovering to 45%. This rapid improvement in hemodynamic parameters and cardiac function allowed VA-ECMO discontinuation after 26 hours of support. The patient was successfully extubated on day 9. RRT was maintained until day 30, when a substantially improved renal function was recorded (creatinine 1.91 mg/dL). Magnetic resonance imaging and electroencephalography performed to evaluate neurological function revealed no evidence of hypoxic-ischemic brain injury. On day 60, the patient was transferred to a rehabilitation facility with intact cognitive function and without neurological sequelae. By this time, the patient's renal function had recovered to within the normal range.

## Discussion

MALA can progress to severe cardiovascular dysfunction, posing a critical therapeutic challenge in its most severe form. On the other hand, knowledge about mechanical circulatory support in severe MALA remains limited [9-11]. Pathophysiological research mainly focuses on mitochondrial dysfunction, associated with metformin-mediated disruption of oxidative phosphorylation in the electron transport chain [12], which leads to lactate accumulation and multi-organ dysfunction. Previous studies have demonstrated that lactate levels and pH values in blood concentration strongly correlate with mortality in patients with MALA [13,14], and higher lactate concentrations are associated with significantly worse outcomes.

Our case demonstrated a successful recovery through the early implementation of VA-ECMO combined with CRRT after cardiac arrest; this strategy challenges the traditional boundaries of survivable cases of severe MALA. Thus, the uniqueness of the present case is associated with survival from an unprecedented metabolic derangement. Currently, RRT is considered the cornerstone of treatment for severe MALA, particularly when lactate levels exceed 20 mmol/L or pH is less than or equal to 7.0. This therapeutic approach facilitates metformin elimination and metabolic acidosis correction [6].

However, mechanical circulatory support with VA-ECMO remains less established in MALA management protocols. While circulatory support devices are typically considered for cardiogenic shock when conventional therapy fails to maintain adequate hemodynamics, several specific criteria guide this decision. These include a cardiac index below 2.2 L•min⁻¹•m⁻² and a requirement for high-dose vasopressors such as norepinephrine exceeding 0.1 μg•kg⁻¹•min⁻¹ despite dobutamine infusion above 5 μg•kg⁻¹•min⁻¹. In addition, persistent lactate levels above 3 mmol/L with a non-decreasing trend or SvO₂ below 50% indicate ongoing tissue hypoperfusion despite adequate filling pressures, further supporting mechanical support consideration [15,16]. Our experience suggests that early consideration of VA-ECMO may be warranted when profound cardiovascular dysfunction persists despite vasopressor and inotropes therapy. Importantly, adequate circulation is essential for effective RRT, making combining these approaches potentially synergistic in severe cases. However, while VA-ECMO provided crucial support in our case, it carries significant risks, including bleeding, limb ischemia, and thrombotic complications, which must be weighed against the benefits. Patient selection for this resource-intensive therapy requires careful consideration of both potential benefits and risks.

The remarkable neurological recovery of our patient deserves particular attention. Extreme metabolic derangements, particularly severe acidosis and hyperlactatemia, are strongly associated with poor neurological outcomes and are often used as criteria for discontinuing resuscitative efforts [17,18]. The initial pH of 6.355 and lactate level of 52.7 mmol/L in our patient represented unprecedented values in surviving MALA cases, values at which neurological recovery would traditionally be considered highly unlikely. Despite these profoundly abnormal parameters that initially suggested an inferior prognosis, our patient achieved complete neurological recovery without any cognitive sequelae. While multiple factors likely contributed to this favorable outcome, including early intervention, the patient's relatively young age with limited comorbidities, and synchronized application of therapeutic modalities, this case challenges conventional prognostic assumptions regarding severe metabolic acidosis. It suggests that with aggressive supportive measures combining VA-ECMO and CRRT, meaningful neurological recovery may be possible even in cases of extreme metabolic derangement that would otherwise be considered unsalvageable.

A distinctive aspect of this case was the development of catastrophic MALA triggered by influenza A infection with subsequent dehydration and acute kidney injury rather than from more commonly reported precipitating factors such as intentional overdose or chronic kidney disease. Despite the patient receiving only maintenance metformin therapy (500 mg daily), metabolic decompensation rapidly progressed, illustrating how common viral illnesses can precipitate severe metabolic crises through altered renal function and impaired lactate clearance [19,20]. Early recognition of MALA in cases of unexplained metabolic acidosis represents a significant diagnostic challenge, particularly since MALA and circulatory failure commonly coexist and complicate each other in critically ill patients. The diagnostic process is further complicated when a complete medication history is initially unavailable, as in this case. Thorough clinical history-taking, even when retrospectively obtained, is crucial in establishing the diagnosis. This case underscores the importance of considering MALA in the differential diagnosis of severe, unexplained lactic acidosis, even when medication history is initially unclear or incomplete. Furthermore, this experience suggests that VA-ECMO may serve as a valuable bridge therapy during the period of diagnostic uncertainty, providing critical organ perfusion and hemodynamic stabilization until a definitive diagnosis is established and targeted interventions can be implemented.

These findings provide crucial insights into managing critical MALA. Although extreme lactate levels and acidosis are often considered for discontinuing resuscitative efforts, our experience suggests that aggressive intervention using VA-ECMO with combined CRRT potentially presents a lifesaving strategy in selected MALA cases, where the underlying pathophysiology of mitochondrial dysfunction can be reversed with adequate support.

## Conclusions

This case suggests that carefully selected patients experiencing MALA-induced profound metabolic derangement and cardiogenic shock may benefit from a combination of VA-ECMO and CRRT support, even in cases presenting with extreme metabolic acidosis. Furthermore, early intervention was a key factor contributing to the successful outcome of our patient. However, these interventions are resource-intensive and carry potential complications that must be weighed against the benefits. Early recognition of MALA in unexplained metabolic acidosis, particularly following acute illnesses such as viral infections and other precipitating factors, can facilitate timely intervention. A coordinated, aggressive approach may offer an effective therapeutic strategy for selected patients, leading to favorable outcomes without neurological sequelae. Future research into optimal timing and patient selection criteria for mechanical circulatory support in severe metabolic emergencies is warranted.
